# The Differential Impact of Medical Therapy and Lifestyle Modification on Cardiovascular Health and Risk of Adverse Cardiovascular Events: A Narrative Review

**DOI:** 10.7759/cureus.57742

**Published:** 2024-04-06

**Authors:** Nikhil Deep Kolanu, Zoya Riyaz Syeda, Nandan Joshi, Prerna Singh, Mounika Erukulla

**Affiliations:** 1 Internal Medicine, China Medical University, Shenyang, CHN; 2 Internal Medicine, Khaja Bandanawaz Institute of Medical Sciences, Gulbarga, IND; 3 Internal Medicine, Surat Municipal Institute of Medical Education and Research, Surat, IND; 4 Internal Medicine, Mayo Clinic, Jacksonville, USA; 5 General Surgery, Osmania General Hospital Affiliated to Osmania Medical College, Hyderabad, IND

**Keywords:** hope, reduce-it, allhat, dash, ldl, cardiovascular disease (cvd)

## Abstract

Cardiovascular diseases (CVDs) continue to be a worldwide health concern, requiring effective strategies for risk reduction. This article explores the extensive collaboration between medical therapy and lifestyle modifications in the management of CVDs, aiming to interpret whether a single approach holds the key to reducing major cardiovascular events. In the realm of pharmaceutical therapy, statins, beta-blockers, angiotensin-converting-enzyme (ACE) inhibitors, and antiplatelet agents have shown significant effectiveness, as evidenced by landmark trials such as Scandinavian Simvastatin Survival Study (4S) and Heart Outcomes Prevention Evaluation (HOPE). Concurrently, lifestyle adjustments, encompassing physical activity, dietary changes, and management of stress, emerge as indispensable elements in cardiovascular care. The article discusses the pivotal role of patient adherence, tailored approaches, and the synergistic impact of combining medical therapy and lifestyle modifications. Challenges, such as socioeconomic disparities and uncertainties in lipid management, underscore the need for ongoing research and precision medicine. Digital health interventions offer novel avenues for personalized care. Despite advancements, uncertainties persist regarding the optimal balance between medical and lifestyle interventions in lowering major cardiovascular event risks. This article emphasizes the ongoing evolution of cardiovascular care, highlighting the imperative need for evidence-based guidelines tailored to individual patient needs.

## Introduction and background

Globally, cardiovascular diseases (CVDs) lead to morbidity and mortality of patients and are a global health concern. CVDs remain the leading cause of death globally, accounting for approximately 17.9 million deaths annually [1]. As medical research advances, the management of CVDs has shown a significant interaction effect between pharmacological interventions and lifestyle adjustments. A broad spectrum of conditions impacting the heart and circulation are included in CVDs. Given the frequency of health risks like diabetes, dyslipidemia, and hypertension, increasing worldwide, the importance of mitigating the impact of CVDs has never been more pressing [2]. The question that resonates in the realm of cardiovascular care is whether one approach, be it medical therapy or lifestyle modifications, holds a singular key to reducing cardiovascular events. This article aims to explore the existing evidence surrounding these two fundamental pillars of cardiovascular management, investigating the areas where pharmaceutical therapies and lifestyle modifications converge.

Medical therapy for CVDs has made significant improvements, with several medicines available targeting different aspects of cardiovascular risk. Angiotensin-converting enzyme (ACE) inhibitors, beta-blockers, statins, and antiplatelet agents constitute formidable options for the prevention and management of CVDs [3]. Investigative studies like the Heart Outcomes Prevention Evaluation (HOPE) project and the Scandinavian Simvastatin Survival Study (4S) have highlighted the potency of these medications in reducing major cardiovascular events and improving overall patient outcomes [4,5]. For instance, the 4S study demonstrated a 30% inhibition in coronary events and a 42% reduction in overall mortality with the use of simvastatin in myocardial infarction patients [4].

Concurrently, lifestyle modifications, nutritional changes, regular physical activity, and weight management appear to be essential elements in the care of cardiovascular health. The INTERHEART study demonstrated that myocardial infarction risk worldwide is accounted for by alterable factors, including smoking, inactivity, and unhealthy diet. [6]. The DASH (Dietary Approaches to Stop Hypertension) trial highlighted the role of eating measures in blood pressure control and CVD risk reduction [7]. Although lifestyle changes and medicinal therapy are sometimes considered separate solutions, their combined effect on lowering cardiovascular risk cannot be understood. The CAMELOT (Comparison of Amlodipine vs. Enalapril to Limit Occurrences of Thrombosis) study demonstrated the additional benefits of lifestyle changes, including a healthy diet and exercise, when combined with medical treatments [8]. The nuances of patient populations, the impact of compliance with prescription drug and lifestyle guidelines, and the long-term sustainability of these interventions warrant further exploration. Addressing these gaps in knowledge is crucial for refining evidence-based guidelines and tailoring cardiovascular care to individual patient needs. This article seeks to unravel the existing evidence, shedding light on whether one approach alone can significantly reduce the chance of significant cardiac events or whether a judicious combination of these two approaches remains the optimal strategy for enhancing cardiovascular health.

## Review

Methodology

Literature Review

Conducting a thorough literature search using databases like PubMed, Medline, and Google Scholar. This review will include studies published with a focus on the relationship between pharmaceutical therapy and lifestyle modifications on cardiovascular health. Key search terms will include "cholesterol," "chronic medical conditions," “Physical therapy” and specific diseases (e.g., diabetes, hypertension). This review will provide a foundation for understanding the existing knowledge and research gaps in this area. Articles were included based on their focus on the clinical presentation, pathophysiology, diagnosis, and management of CVDs, considering both original research and review articles. Data extracted from the selected articles were synthesized to provide a detailed overview of the current understanding, highlighting key findings and controversies. This review specifically addressed the management strategies and risk stratification. While efforts were made to conduct a comprehensive review, limitations inherent to narrative reviews should be considered.

Inclusion criteria: Adult individuals up to 70 years and the presence of a minimum of one risk factor for CVDs (e.g., hypertension, dyslipidemia, diabetes, smoking).

Exclusion criteria: Known intolerance or contraindications to medical therapy or lifestyle modifications.

Intervention

Medical therapy group (MTG): Participants assigned to the MTG were prescribed standard pharmacological treatments as per current cardiovascular disease management guidelines, with prescription adjustments made as necessary depending on individual response and side effects.

Lifestyle modification group (LMG): Participants assigned to the LMG received counseling and education on lifestyle adjustments, such as food adjustments and increased exercise, smoking cessation techniques, and stress management strategies, tailored to their individual risk profiles.

Discussion

A detailed discussion of each of the intervention methodologies is presented below.

Medical Therapy for CVDs

Clinical guidelines and pharmacological medications play an essential role in the management of CVD. Medications targeting specific risk factors have been demonstrated to be successful in lowering the morbidity and death linked to CVDs.

Cholesterol management: Cholesterol management is a critical component of overall cardiovascular health, as elevated blood cholesterol causes the accumulation of plaque in the arteries, raising the possibility of cardiovascular disease and stroke. While lifestyle changes including eating a balanced diet and doing frequent exercise are essential in managing cholesterol levels, medical therapy can play a crucial role, especially in individuals with markedly high cholesterol or those at heightened risk of developing CVD.

Statins, such as atorvastatin and rosuvastatin, have proven efficacy in lowering LDL lowering blood cholesterol and lowering the chance of atherosclerotic events [9]. It has been demonstrated that statins can lower the risk of significant cardiac events by as much as 30% in patients with hyperlipidemia [10]. Statins function by preventing the synthesis of cholesterol by suppressing HMG-CoA reductase in the liver. By reducing cholesterol synthesis, statins not only lower LDL (low-density lipoprotein) levels but also have other pleiotropic effects, such as reducing inflammation and stabilizing plaques.

PCSK9 inhibitors, such as evolocumab and alirocumab, offer additional LDL reduction for high-risk patients [11]. They inhibit an enzyme that degrades LDL receptors and has also emerged as an effective treatment option in patients with resistant hypercholesterolemia or those with familial hypercholesterolemia.

Ezetimibe, as an adjunct to statins, provides incremental benefits in cholesterol control [12]. It is a cholesterol absorption inhibitor and can be used with statin treatment to further lower LDL cholesterol levels. Lipid-lowering drugs and their effects are shown in Table 1.

**Table 1:** Common lipid-lowering drugs and their effects.

Class	Examples	Effect on Lipid Profile
Statins (HMG-CoA reductase inhibitors)	Rosuvastatin, Atorvastatin, Lovastatin, Simvastatin Pravastatin	↓ LDL, ↓ Triglycerides
Niacin	Niaspan	↑ HDL, ↓ LDL
Fibrates (Lipoprotein lipase stimulators)	Gemfibrozil	↓ Triglycerides, ↑ HDL.
Bile acid Resins	Cholestyramine, Colestipol, Colesevelam	↓ LDL
Proprotein convertase subtilisin/ kexin type 9 (PCSK9) inhibitors	Alirocumab, Evolocumab,	↓ LDL
Cholesterol absorption inhibitors (Ezetimibe)	Ezetimibe	↓LDL

Blood pressure (BP) control: BP control is critical for maintaining cardiovascular health and preventing complications such as renal failure, heart disease, and stroke. While lifestyle changes including stress management, regular exercise, and eating a nutritious diet reduction technique are fundamental in managing BP, medical therapy is often necessary, especially for individuals with hypertension who are difficult to control or who possess an elevated probability of heart-related incidents.

Antihypertensive medications, including ACE inhibitors (e.g., enalapril) and beta-blockers (e.g., metoprolol), contribute to BP regulation, reducing strain on the cardiovascular system [13]. These medications act through different mechanisms to reduce BP and the workload on the heart, ultimately lowering the possibility of unfavorable cardiovascular events.

Extensive trials in medicine have demonstrated the efficacy of antihypertensive medications. The ALLHAT trial compared four major types of medications calcium channel blocking drugs, ACE inhibitors, and diuretics such as thiazide and alpha-blockers are used to treat hypertension, in more than 42,000 participants with hypertension [14]. The study showed that diuretics, specifically chlorthalidone, not only were effective at lowering BP but also reduced heart failure risk and stroke. The HOPE study investigated the impact of ramipril, an ACE inhibitor, in those who are at high risk of cardiovascular events with or without hypertension. This trial revealed a notable decline in the frequency of myocardial infarction stroke, and death from cardiovascular disease among ramipril users. The valsartan and amlodipine were examined in the VALUE study, in more than 15,000 high-risk hypertensive patients [15]. Both treatment groups achieved similar BP reductions, but the possibility of cardiac arrest and stroke is lower in the group taking valsartan.

Antiplatelet therapy: Antiplatelets are a crucial part of treatment for CVDs. Platelets play a crucial role in the formation of blood clots, and excessive platelet aggregation can lead to the development of thrombotic incidents like heart attack or stroke. Antiplatelet drugs are designed to inhibit platelet activation, preventing the formation of harmful blood clots. In addition to aspirin, several other antiplatelet drugs created with the intention of treating cardiovascular illnesses. One such class of drugs is P2Y12 receptor inhibitors, which block the action of adenosine diphosphate (ADP) on platelet aggregation. Examples of P2Y12 receptor inhibitors include clopidogrel, ticagrelor, and prasugrel. These drugs are often used as well as aspirin to offer dual antiplatelet therapy, further reducing the risk of clot formation. The prodrug clopidogrel needs to be activated by liver enzymes to produce antiplatelet effects. Ticagrelor and prasugrel are direct-acting inhibitors that do not require hepatic activation. Studies have shown that compared to clopidogrel, ticagrelor and prasugrel lead to better clinical results for those suffering from acute coronary syndrome.

Aspirin and P2Y12 inhibitors (e.g., clopidogrel) are cornerstone antiplatelet agents that prevent thrombotic events among individuals with a history of myocardial infarction or stroke [16].

Another emerging therapy in antiplatelet treatment is the use of protease-activated receptor 1 (PAR-1) inhibitors. Vorapaxar is a PAR-1 antagonist that prevents the activation of platelets by thrombin. It has been demonstrated to lower the chance of heart-related incidents. when used in conjunction with standard antiplatelet treatment in individuals with previous experiences of peripheral arterial disease or myocardial infarction [17]. Although antiplatelet therapy is efficient in lowering heart attacks, it is not without risk. The most significant adverse effect associated with antiplatelet drugs is bleeding, particularly in the gastrointestinal tract or intracranially. The risk of bleeding should be carefully evaluated before initiating therapy, and the benefits of using antiplatelet treatment should outweigh the potential risks.

Lifestyle Modifications in CVDs

Complementary to medical therapy, lifestyle modifications are fundamental for comprehensive cardiovascular risk reduction. These changes address modifiable risk factors and empower individuals to actively participate in their cardiovascular health.

Dietary interventions: Adopting heart-healthy food habits, like DASH or the Mediterranean diet, highlights the consumption of vegetables, fruits, and entire grains while limiting saturated fat and sodium intake. Clinical trials have shown that following the DASH diet can significantly reduce BP levels, making it an effective intervention for individuals with hypertension. Several studies, such as the PREDIMED trial [18], showed that following the Mediterranean food regimen is connected with a lower risk of heart disease and stroke. A meta-analysis of cohort studies discovered that following a nutritious diet was associated with a 20% decreased risk of getting coronary heart disease [19].

Diets based on plants, such as vegan and vegetarian diets, have become increasingly common because of their possible health advantages. These diets are centered around plant foods, including fruits, vegetables, legumes, nuts, and entire grains while excluding or minimizing the consumption of animal products. Plant-based diets have been shown in studies to lower the possibility of cardiac illnesses, likely through favorable BP-related effects, cholesterol levels, and inflammation [20]. The American Heart Association suggests cutting the daily allowance of sodium by no more than 2,300 milligrams, and for those who already have hypertension or are at high risk of getting it, to 1,500 mg [21]. Studies, such as the REDUCE-IT trial, have demonstrated Omega-3 fatty acids and their cardioprotective properties [22].

Physical activity: Regular physical exercise is essential for controlling and preventing cardiovascular diseases. There are several advantages to engaging in routine physical activity, such as reducing the possibility of heart attack, stroke, and other cardiovascular-related complications. It also helps keep your BP and levels of cholesterol in check, keep weight in a healthy range, improve insulin sensitivity, and promote overall cardiovascular health.

Adults should perform aerobic exercise lasting at least 150 minutes every week with a moderate intensity or 75 minutes each week at a strong intensity, according to the American Heart Association's recommendations, with muscle-strengthening exercises added in at least twice per week [23]. Sports, aerobics, and brisk walking were considered vigorous-intensity activities, whereas swimming and cycling were considered moderate-intensity.

Several studies have shown the benefits that exercise provides on cardiovascular health. A meta-analysis by Cornelissen demonstrated a lower probability of cardiovascular death among those who regularly exercised [24]. Moreover, exercising has several advantages for managing and controlling several risk factors that contribute to CVD. Regular exercise helps reduce BP readings and increase the overall circulation of blood [25]. It also aids in maintaining healthy cholesterol levels via raising HDL cholesterol, which is considered beneficial for cardiovascular health [26]. Being physically active also plays a crucial influence in preventing and managing obesity, which is a recognized contributory factor for heart conditions [27]. Furthermore, regular physical activity can enhance sensitivity to insulin and regulate glucose, which are crucial for halting the development of diabetes type 2, which is a significant cardiovascular risk factor [28].

Smoking cessation: Smoking cessation is a critical lifestyle modification for individuals with cardiovascular diseases. Smoking is an acknowledged agent of risk for the development and advancement of several cardiovascular disorders, notably stroke, coronary artery, and peripheral artery disease. It has been demonstrated that stopping the habit of smoking significantly lowers the chance of developing certain cardiovascular illnesses and enhances general cardiovascular health. For example, the publication of a meta-analysis in the journal Circulation showed that quitting smoking after a heart attack reduces the chance of recurrent cardiovascular incidents by more than 35% [29]. An additional investigation that was published in the New England Journal of Medicine showed that even long-term smokers who quit smoking at the age of 35 effectively reduce their chance of death because of heart disease [30]. A meta-analysis by Critchley & Capewell showed a 36% decrease in the risk of cardiovascular events [31].

There are various strategies and interventions available to help individuals quit smoking. These include counseling, behavioral therapies, and pharmacological treatments. The using nicotine substitution therapy products, like gum or patches, has been shown to be effective at reducing nicotine cravings and signs of withdrawal when trying to stop smoking [32].

Stress management: Stress management is an important aspect of lifestyle modifications for individuals with CVDs. Chronic stress was linked to a higher chance of developing and exacerbating cardiovascular conditions, like hypertension and coronary artery disease. Implementing effective stress management techniques can improve cardiovascular health and lower the risk of adverse outcomes.

Numerous studies have highlighted the influence of chronic stress on cardiovascular health. A meta-analysis by Rosengren et al. demonstrated that individuals with high levels of job strain, a significant source of stress, had a greater risk of developing CHD [33]. There are various stress management techniques that can be implemented to promote cardiovascular health. One commonly recommended method is relaxation therapy, which includes techniques include progressive muscular relaxation, meditation, and deep breathing. It has been demonstrated that relaxation treatments lower BP, heart rate, and stress levels overall [34].

Cognitive-behavioral therapies (CBTs) can also work well at managing stress. The objective of CBTs is to identify and alter negative thought patterns and stress-related behaviors. Several studies have shown the beneficial effects of CBTs in reducing stress levels and improving psychological well-being in individuals with cardiovascular diseases [35]. Regular physical activity, as mentioned earlier, not only contributes to overall cardiovascular health but also serves as an effective stress management strategy. Engaging in aerobic exercise can help alleviate stress and improve mood through endorphin release, commonly referred to as "feel-good" hormones [36].

Interaction Between Medical Therapy and Lifestyle Modifications

The synergy between medical therapy and lifestyle modifications is paramount in achieving ideal outcomes in cardiovascular care. While medications effectively target specific risk factors, lifestyle modifications address the broader spectrum of modifiable risks, promoting sustained cardiovascular health.

Patient adherence: Addressing barriers to adherence, such as medication side effects, cost, and access to healthy foods or exercise facilities, is crucial. Healthcare providers should work closely with patients to overcome these barriers, such as by switching medications to those with fewer side effects or providing resources and referrals to affordable healthy food options or exercise programs. Furthermore, involving patients in shared decision-making can also enhance adherence. By involving individuals in the process of making decisions, medical professionals can customize treatment regimens to meet personal preferences, values, and lifestyles, leading to greater motivation and ownership of the treatment regimen.

Patient adherence to prescribed medications and lifestyle changes are integral to success. Healthcare providers play a crucial role in patient education and ongoing support [37].

Tailored approaches: Tailored approaches that involve the combination of medical therapy and lifestyle modifications has demonstrated to be more successful at achieving optimal results.

Several studies have shown the advantages of combining medical therapy with lifestyle modifications. In a study by Menezes et al. [38], a comprehensive cardiac rehabilitation program, which encompassed both medical therapy and lifestyle modifications, was shown to significantly reduce the risk of hospital readmission and mortality in patients with coronary artery disease. Another study by Kotseva et al. revealed that a combination of pharmacotherapy with lifestyle measures, such as dietary modifications and exercise, had synergistic effects on improving lipid profiles and reducing overall cardiovascular risk [39].

Tailored approaches that consider the individual patient's needs, preferences, and capabilities are essential for successful implementation of lifestyle modifications. Healthcare providers should assess patients’ readiness for change, identify barriers to lifestyle modifications, and develop personalized strategies to overcome these barriers. For example, in patients with obesity and cardiovascular diseases, a tailored approach may involve a combination of dietary counseling, physical activity recommendations, and weight management strategies based on the patient's preferences and abilities [40]. Moreover, regular follow-up and monitoring of patients are crucial for ensuring adherence to lifestyle modifications and optimizing medical therapy. Healthcare providers should provide ongoing support, education, and counseling to motivate and empower patients to sustain lifestyle changes. In a study, it was found that regular telephone support by healthcare professionals was effective at improving adherence to lifestyle modifications and decreasing cardiovascular incidents in people who have a history of coronary artery disease [41]. An overview of medical therapy and lifestyle modifications is shown in Table 2.

**Table 2:** An overview of medical therapy and lifestyle modifications.

Aspect	Medical Therapy	Lifestyle Modifications
Cholesterol Management	Medications like statins, PCSK9 inhibitors, and ezetimibe can lower LDL cholesterol, lowering the chance of cardiovascular events and atherosclerosis.	Adoption of heart-healthy diets, regular exercise, and weight management contribute to improved cholesterol profiles and cardiovascular health.
Blood Pressure Control	Antihypertensive medications assist in blood pressure regulation, lowering the risk of heart attack and stroke, and other cardiovascular events.	Dietary sodium reduction, increased controlling weight and engaging in physical activity help lower blood pressure and cardiovascular risk reduction.
Antiplatelet Therapy	Aspirin and other antiplatelet medications reduce the risk of blood clot formation, lowering the risk of cardiovascular events.	Adherence to medications is crucial for the effectiveness of antiplatelet therapy. No specific lifestyle modifications directly affect this aspect.
Dietary Interventions	Medications may address lipid profiles, but dietary changes are essential for long-term management and prevention of cardiovascular events.	Adopting heart-healthy diets (Mediterranean, DASH) high in fruits, vegetables, and whole grains is advised to lower the incidence of significant heart-related incidents.
Physical Activity	Medications focus on specific risk factors; regular exercise enhances cardiovascular fitness and reduces the risk of cardiovascular events.	Engaging in regular exercise supports overall heart health and lowers risk of major cardiovascular events.
Smoking Cessation	Medications aid in quitting smoking, decreasing the chance of heart-related incidents.	Quitting smoking is a critical lifestyle change with immediate and profound cardiovascular benefits, significantly reducing the risk of major events.
Stress Management	Medications may help manage stress-related conditions, but lifestyle approaches are crucial for long-term stress reduction and cardiovascular risk reduction.	Techniques like mindfulness, relaxation, and exercise contribute to stress reduction and cardiovascular event prevention.
Weight Management	Medications for weight loss may be prescribed, but healthy eating habits and regular exercise play a central role in cardiovascular event prevention.	Maintaining a healthy weight through lifestyle changes is crucial for reducing the chance of serious heart-related incidents.
Diabetes Management	Antidiabetic medications aim to control blood sugar levels, lowering the chance of heart-related incidents.	Lifestyle changes, including a balanced diet and regular exercise, play a central role in managing diabetes and preventing major cardiovascular events.
Adherence and Patient Education	Healthcare providers serves a crucial part in educating patients and promoting adherence to medications, reducing the risk of cardiovascular events.	Patient education on healthy lifestyles, ongoing support, and addressing barriers to change are crucial for sustained lifestyle modifications and cardiovascular risk reduction.
Overall Cardiovascular Risk Reduction	Medications address specific risk factors, contributing to overall cardiovascular risk reduction.	Lifestyle modifications contribute to a broader spectrum of modifiable risks, promoting overall cardiovascular health and reducing the risk of major events.
Individualized Approaches	Tailored medication regimens based on patient profiles.	Personalized lifestyle recommendations based on individual needs, preferences, and cultural considerations.

Challenges and Future Directions

Despite the aforementioned advancements, challenges persist in translating these strategies into widespread, sustained cardiovascular health improvements. Barriers to lifestyle modifications, socioeconomic disparities, and potential side effects of medications underscore the need for ongoing research and community-based interventions.

Precision medicine: One of the major challenges in the application of precision medicine in cardiovascular diseases is the identification of relevant genetic and molecular markers. Although there have been significant advances in genomics and the ability to sequence the entire human genome, the complexity of CVD makes it challenging to identify specific genetic variations or biomarkers that can accurately predict disease risk or treatment response. Additionally, cardiovascular numerous hereditary and environmental variables frequently have an impact on diseases, making it challenging to pinpoint the exact mechanisms underlying disease pathogenesis.

Furthermore, the development of precision therapeutics, such as gene editing technologies and targeted drug delivery systems, could greatly enhance treatment options for CVD. Advances in gene editing, such as CRISPR‒Cas9, hold the potential to correct genetic mutations that contribute to CVD. Targeted drug delivery systems can increase the efficacy and reduce the side effects of cardiovascular medications by specifically targeting affected tissues or cells. Research on precision medicine aims to tailor medical therapies based on genetic and molecular profiles, potentially leading to more targeted and effective treatments [42].

Digital health interventions: There are several future directions that hold promise for the advancement of precision medicine in cardiovascular diseases. The combination of machine learning algorithms and artificial intelligence (AI) is among the main areas of focus. These technologies can help analyze large datasets, identify disease patterns, and generate personalized treatment recommendations. AI algorithms can also help predict disease outcomes and stratify patient populations, enabling more targeted and effective interventions.

The integration of digital health tools, including mobile applications and wearable devices, provides novel avenues for monitoring patient adherence and offering real-time feedback and support [43]. A review of some key trials in medical intervention and lifestyle modifications, their objectives, and key findings is presented in Table 3.

**Table 3:** Review of some key trials in medical intervention and lifestyle modifications, their objectives, and key findings. HOPE: Heart Outcomes Prevention Evaluation; ALLHAT: The Antihypertensive and Lipid-Lowering Treatment to Prevent Heart Attack Trial; VALUE: Valsartan Antihypertensive Long-term Use Evaluation; DASH: Dietary Approaches to Stop Hypertension;

Trials	Objectives	Key findings
HOPE trial [4]	Test the theories that, when compared to a placebo, two preventative intervention strategies— Individuals who are at a greater chance of cardiovascular events would experience less morbidity and death with ACE inhibition or vitamin E.	Management via ramipril minimized the prevalence of fatalities from cardiovascular causes (6.1%, as compared with 8.1% in the placebo group; relative risk, 0.74; P<0.001), myocardial infarction (MI) (9.9% vs. 12.3%; relative risk, 0.80; P<0.001), a stroke (3.4% vs. 4.9%; relative risk, 0.68; P<0.001), passing away from any reason (10.4% vs. 12.2%; relative risk, 0.84; P=0.005), the procedures of revascularization (16.3% vs. 18.8%; relative risk, 0.85; P<0.001), cardiac arrest (0.8% vs. 1.3%; relative risk, 0.62; P=0.02), heart failure (9.1% vs. 11.6%; relative risk, 0.77; P<0.001), and issues associated with diabetes (6.4% vs. 7.6%; relative risk, 0.84; P=0.03).
ALLHAT trial [13]	The lipid lowering and Antihypertensive treatment to Prevent Heart Attack	In compared to chlorthalidone (6-year rate, 11.5%), the relative risks (RRs) for amlodipine (6-year rate, 11.3%) and lisinopril (6-year rate, 11.4%) were 0.98 (95% CI, 0.90-1.07) and 0.99 (95% CI, 0.91-1.08), respectively. There was also no difference in the all-cause death rate between the groups. Amlodipine had a significantly lower five-year diastolic blood pressure (0.8 mm Hg, P<.001 when compared to chlorthalidone whereas the five-year systolic blood pressure mm hg p=".03)" and lisinopril groups had significantly higher pressure. relative risks for rate amlodipine were ci respectively in comparison rates of deaths from all causes did not differ either. comparing drug caused a substantial drop diastolic while taking considerably
VALUE trial [14]	Valsartan or amlodipine effect on cardiovascular risk	Both therapies lowered blood pressure, although amlodipine benefits had been more noticeable, particularly in the early going (blood pressure was 4.0/2.1 mm Hg lower in the amlodipine group after one month and 1.5/1.3 mm Hg lower in the valsartan group after one year; p<0.001 between groups). 810 patients (10.6%, or 25.5 per 1000 patient-years) treated with valsartan, whereas 789 patients (10.4%, or 24.7 per 1000 patient-years) treated with amlodipine (hazard ratio 1.04, 95% CI 0.94-1.15, p=0.49). experienced the main composite outcome.
DASH trial	Relationship between blood pressure and food habits	Compared to the control diet, the fruits and vegetables diet reduced BP by 1.1 mm Hg (P=0.07) and 2.8 mm Hg more in the diastolic range (P<0.001). Compared to the control diet, the combination diet decreased systolic and diastolic blood pressure by 5.5- and 3.0-mm Hg more, respectively (P<0.001 for each). In the 326 subjects with no hypertension, the associated reductions were 3.5 mm Hg (P<0.001) and 2.1 mm Hg (P=0.003). The combination diet decreased both systolic and diastolic blood pressure by 11.4- and 5.5-mm Hg more, respectively, than the control diet (systolic pressure, > or = 140 mm Hg; diastolic pressure, > or = 90 mm Hg; or both).
PREDIMED trial [17]	There is a connection between heart disease risk and following the Mediterranean diet.	The hazard ratio for a Mediterranean diet including extra-virgin olive oil was 0.69 (confidence interval, 95% [CI], 0.53 to 0.91) compared to the control diet, and it was 0.72 (0.54 to 0.95, 95% CI) for a Mediterranean diet including nuts.

## Conclusions

As we explore the challenging areas of CVD management, the contrast between medical therapy and lifestyle modifications creates a vital discussion. In conclusion, the dynamic integration of medical therapy and lifestyle modifications is imperative for effective CVD management. The intricate interplay between these modalities demands a holistic, patient-centered approach that acknowledges the uniqueness of each individual's cardiovascular risk profile. As the field advances, ongoing research, innovation, and a commitment to fostering healthier communities will be instrumental in the pursuit of a world with reduced cardiovascular burdens.

The existing scientific consensus underscores the complex nature of cardiovascular health. Ultimately, the dichotomy emphasizes the importance of comprehensive cardiovascular risk management, urging individuals to be proactive in adopting healthy lifestyles and seeking professional guidance for personalized care. As research continues to unravel the complexities of cholesterol dynamics, a holistic approach to cardiovascular health remains the cornerstone for a thriving heart.
